# Young Patient Presenting Acute Coronary Syndrome

**DOI:** 10.14740/jocmr1971w

**Published:** 2014-10-16

**Authors:** Sungbae Ju, Hee-Sun Mun, Seonghoon Choi, Jung Rae Cho, Namho Lee, Min-Kyung Kang

**Affiliations:** a Cardiology Division, Kangnam Sacred Heart Hospital, Hallym University Medical Center, Seoul, Korea

**Keywords:** Kawasaki disease, Coronary aneurysm, Acute coronary artery

## Abstract

The clinical presentation of Kawasaki disease (KD) is variable and clinical implication among adults is rarely important but coronary involvement. Here we report a young patient showing recurrent acute coronary syndrome (ACS) who had a history of high-grade fever and conjunctivitis when he was little. Coronary angiography revealed aneurysmal coronary artery change in this patient. There is no particular consensus on guidelines for treatment for KD in case of coronary aneurysm causing ACS. In this case, we treated him medically without stent implantation successfully.

## Introduction

Acute coronary syndrome (ACS) is the clinical presentation of myocardial ischemia. Patients aged 35 years or less account for only a minor proportion of all patients with ACS. Only few studies have focused on the clinical presentation, treatment and outcome of ACS in young patients, and many of them were restricted to specific patient population [1]. Among them, Kawasaki disease (KD) is one of the important causes for ACS among young patients aged 35 or less, because of coronary involvement. Coronary artery abnormalities that can affect individuals late after KD include persistent aneurysms with the risk of thrombosis and progressive stenosis, with or without the development of extensive collateral circulation [2]. There are few reports of coronary involvements after KD that occurred in young patients; however, there are no confirmed treatment guidelines for those particular patient groups. Therefore, here we report a case of ACS in a young patient who was suspected of coronary aneurysm after KD and treated with antiplatelet therapy successfully.

## Case Report

**Figure 1:**
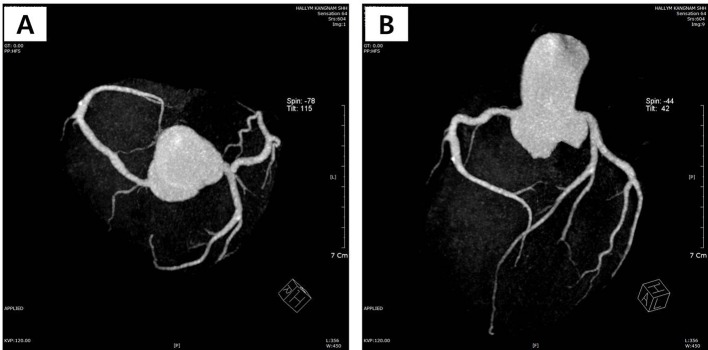
(A, B) Coronary computed tomography showing normal coronary arteries without stenosis.

**Figure 2:**
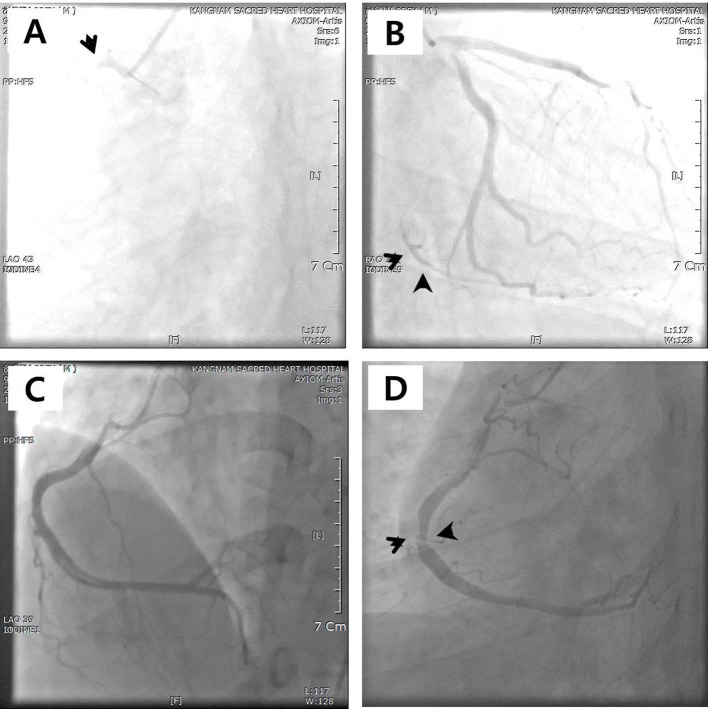
Coronary angiography (CAG) at the time of acute coronary syndrome. (A) Complete obstruction of proximal right coronary artery (RCA). (B) Restoration of RCA flow after angioplasty and thrombus suction. (C, D) CAG at the time of recurred angina showing stenosis of distal right coronary artery with aneurysmal change.

A 36-year-old male presented with chest pain that had lasted for 1 week. He was a 45 pack-years current smoker and previously he was diagnosed mild fatty liver disease and minimal coronary artery disease by coronary computed tomography (CT) 1 year ago (Fig. 1) and recommended for life style modification. Initial electrocardiography showed normal sinus rhythm without ST change and transthoracic echocardiography showed normal left ventricular systolic function without regional wall motion abnormality. However we performed coronary artery angiography (CAG) because troponin I was mildly elevated (0.061 ng/mL). CAG revealed total occlusion of proximal right coronary artery (RCA) by heavy thrombus (Fig. 2A). We did balloon angioplasty and suction of thrombus with restoration of RCA flow (Fig. 2B). He did not have any other conventional risk factors for coronary artery disease (CAD) except smoking. All laboratory parameters were all normal including inflammatory and autoimmune markers. He was discharged with anti-anginal medications with good adherence. After 7 months, his chest pain and dyspnea on exertion relapsed. We again performed CAG which revealed significant stenosis of distal RCA (Fig. 2C, D). We did not perform stent implantation because proximal diameter of the lesion was more than 6 mm; instead we continued intensive medical treatments. We have come to suspicion of KD when putting his clinical aspects together: ACS occurred at young age with only smoking and history of high-grade fever and conjunctivitis when little.

## Discussion

KD was first described in Japan by Dr. Tomosaku Kawasaki in 1967 and originally called mucocutaneous lymph node syndrome [3]. The clinical presentation of KD is variable, but classically consists of fever, accompanied by nonprurent conjunctivitis, generalized exanthem, oral-mucosal and extremity changes, and cervical lymphadenopathy [4]. Coronary aneurysms, the hallmark feature of KD, which form during the acute phase of KD can remodel, remain unchanged, progress to stenotic lesions, or thrombus, which can cause acute myocardial infarction among young adulthood [2]. In the general adult population, less than 10% of patients are under 50 years of age when they develop ACS; however, about 90% of patients experienced ACS when less than 40 years old [5]. Giant calcified aneurysms involving the proximal portion of the major branches were the most common culprit lesions found, and in most cases thrombus formation within the aneurysm precipitated the ACS [5]. Likewise, thrombus formation was the cause of total occlusion of RCA in this patient and aneurysmal change of RCA was detected later in this case even though not giant (> 8 mm).

Treatment options consist of surgical, percutaneous, and medical approaches. Despite many issues of coronary aneurysms, there are still no guidelines for therapeutic management. Aggressive management of traditional cardiovascular risk factors and medical therapy using anticoagulation or antiplatelet therapy and/or statin is recommended because of an increased thrombotic risk in patients with aneurismal arteries [3]. In addition, even if the aneurysms regress, the risk of ACS remains in adult life. Smoking was a prominent additional risk factor [5] like our patient. Therefore, smoking cessation is strongly recommended to prevent further event.
